# Chronic Cerebral Hypoperfusion Aggravates Parkinson's Disease Dementia-Like Symptoms and Pathology in 6-OHDA-Lesioned Rat through Interfering with Sphingolipid Metabolism

**DOI:** 10.1155/2022/5392966

**Published:** 2022-08-08

**Authors:** Yaohua Fan, Mengzhu Li, Chunxiao Wu, Yubiao Wu, Jiajun Han, Peipei Wu, Zifeng Huang, Qizhang Wang, Lijun Zhao, Dongfeng Chen, Meiling Zhu

**Affiliations:** ^1^Shenzhen Hospital of Integrated Traditional Chinese and Western Medicine, Shenzhen, China; ^2^Guangzhou University of Chinese Medicine, Guangzhou, China; ^3^Basic Medical College, Guangzhou University of Chinese Medicine, Guangzhou, China

## Abstract

Chronic cerebral hypoperfusion (CCH) is a cardinal risk factor for Parkinson's disease dementia (PDD), but this potential causality lacks mechanistic evidence. We selected bilateral common carotid artery occlusion (BCCAO) to simulate chronic cerebral hypoperfusion in the rat model of PD induced by typical neurotoxin 6-hydroxy dopamine (6-OHDA). Four weeks after unilateral injection of 6-OHDA into the medial forebrain bundle, rats underwent BCCAO. Male Sprague-Dawley rats were divided into five groups of ten, including sham, PD+BCCAO 2 weeks, PD+BCCAO 1 week, PD, and BCCAO 2 weeks. Then, open field test (OFT) and *Morris* water maze test (MWM) were used to assess the PDD-like symptoms in rats. Also, the pathological manifestations and mechanisms of BCCAO impairing cognitive functions have been explored via hematoxylin-eosin staining, Nissl staining, immunohistochemistry, immunofluorescence, RNA sequencing analysis, lipidomics, and quantitative real-time polymerase chain reaction. In this study, we found that CCH could aggravate PDD-like cognitive symptoms (i.e., learning memory and spatial cognition) and PDD-like pathology (higher expression of *α*-Syn and A*β* in prefrontal cortex and striatum). Moreover, a potential relationship between differentially expressed mRNAs and lipid metabolism was revealed by RNA sequencing analysis. Lipidomics showed that CCH could affect the intensity of 5 lipids, including sphingomyelin (SM 9:0;2O/26:2; SM 8:1;2O/25:0; and SM 8:0;2O/28:4), cardiolipin, lysophosphatidylcholine, cholesteryl ester, and triacylglycerol. Interestingly, the KEGG pathway analysis of both RNA sequencing analysis and lipidomics suggested that CCH leaded to learning impairment by affecting sphingolipid metabolism. Finally, we found that CCH disrupts the sphingolipid metabolism by affecting the mRNA expression of SMPD1 and SMS2, leading to the accumulation of sphingomyelin in the prefrontal cortex. In summary, CCH, an independent exacerbating reason for impairment in learning and memory within the pathopoiesis of PD, aggravates Parkinson's disease dementia-like symptoms and pathology in 6-OHDA-lesioned rat through interfering with sphingolipid metabolism.

## 1. Introduction

Cognitive impairment is one of the most prominent and disabling nonmotor symptoms of Parkinson's disease (PD) [1]. At present, in PD patients with a course of 10 years or more, the cumulative prevalence of Parkinson's disease dementia (PDD) is 75-90% [2]. With increasing life expectancy of PD patients, PDD is set to become even more prevalent in the future. It would seriously affect the patient's quality of life. The etiology of PDD involves genetics, neurophysiology, neuropathology, neuroimaging, environment, vascular factors, and other aspects of research [3]. On the one hand, a growing number of studies showed that synergistic effects of alpha-synuclein (*α*-Syn) and beta-amyloid (A*β*) are closely associated with PDD severity [4, 5]. *α*-Syn inclusion formation could impair cognitive behaviors associated with functions of prefrontal cortex [6]. On the other hand, brain magnetic resonance imaging (MRI) studies showed white matter hyperintensities and a postural drop in blood pressure among PD patients, which revealed recurrent episodic hypotension results in cerebral hypoperfusion (CH), in turn causing anoxic damage to vulnerable areas of the brain and impaired cognitive function [7, 8]. Several studies found that chronic cerebral hypoperfusion (CCH) independently exacerbated cognitive impairment in 1-methyl-4-phenyl-1,2,3,6-tetrahydropyridine- (MPTP-) lesioned mice via impairing microvascular in the hippocampus and white matter [9, 10]. Despite the efforts to improve the knowledge pertaining the pathological bases of PDD, the contribution of CCH is still unclear. Therefore, in-depth study of the mechanism of CCH in the occurrence and development of PDD will help to improve the pathological understanding of PDD and promote the development of new treatment methods for PDD.

Bilateral common carotid artery occlusion (BCCAO) is a common method to induce CCH in rat [11, 12]. Therefore, we selected BCCAO to simulate chronic cerebral hypoperfusion in the rat model of PD induced by typical neurotoxin 6-hydroxy dopamine (6-OHDA) [3, 13, 14]. Then, we explored the autonomic motor and cognitive functions of those rats through open field test (OFT) and *Morris* water maze test (MWM). Furthermore, the pathological manifestations and mechanisms of BCCAO impairing cognitive functions have been explored via immunohistochemistry (IHC), immunofluorescence (IF), ribonucleic acid (RNA) sequencing analysis, lipidomics, and quantitative real-time polymerase chain reaction (qRT-PCR). Our results would provide new ideas for the study of mechanism of CCH enhancing PDD-like symptoms and pathology.

## 2. Materials and Methods

### 2.1. Animal

**Figure 1:**
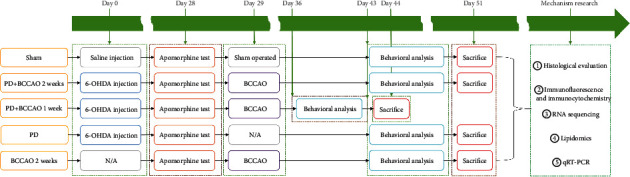
The timeline of the experiment. Abbreviations: PD: Parkinson's disease; BCCAO: bilateral common carotid artery occlusion; 6-OHDA: 6-hydroxy dopamine; N/A: no operation; RNA: ribonucleic acid; qRT-PCR: quantitative real-time polymerase chain reaction.

Male Sprague-Dawley rats (200-220 g) were purchased from Experimental Animal Center of Guangzhou University of Chinese Medicine (Guangzhou, China) and maintained in cages under standard conditions with a 12 h light/dark cycle with free access to food and water. All experiments were carried out in accordance with the Guide for the Care and Use of Laboratory Animals and were approved by the Ethics Committee for Animal Research of Guangzhou University of Chinese Medicine (No. 20210318006). For all experiments, rats were acclimated to their surroundings for 3 days in the animal room prior to surgery, and they had free access to food and water. Then, rats were divided into five groups of ten. Body weight was measured once a week. The intervention methods of rats in each group are shown in Figure 1.

### 2.2. Unilateral Injection of 6-Hydroxy Dopamine into the Medial Forebrain Bundle (MFB)

The rat model of PD was established by unilateral injection of 6-hydroxy dopamine (6-OHDA) into MFB as previously described with minor modifications [15]. Rats were anaesthetized with isoflurane and treated with carprofen as an analgesic. Also, rats are prone to death due to hypothermia during anesthesia. Thus, surgery was carried on a heating pad to prevent hypothermia. Rat skulls were exposed and then drilling a hole in the right side of the MFB (2.2 mm posterior to the anterior fonticulus, 1.5 mm on the right side of the center line, and 8.0 mm subdural). Subsequently, 4 *μ*L of 6-OHDA (MedChemExpress, USA) solution (5 *μ*g/*μ*L; dissolved in saline containing 2 mg/mL ascorbic acid) was injected into the right MFB at a rate of 1 *μ*L/min. Rats injected with the same volume of saline containing 2 mg/mL ascorbic acid were used as the sham group. Apomorphine (Wako, Japan) solution (0.05 mg/kg, dissolved in saline containing 0.02% ascorbic acid) was subcutaneously used to induce the spontaneous rotational movement in rat 4 weeks after model establishment and before sacrificing. The number of rotations contralateral to the lesion during 30 min after apomorphine injection was evaluated [16].

### 2.3. Bilateral Common Carotid Artery Occlusion Surgery

Rats were anaesthetized with isoflurane and treated with carprofen as an analgesic. Also, surgery was carried on a heating pad to prevent hypothermia. BCCAO was performed as described in the literature [17]. A midline ventral cervical incision was made to expose both common carotid arteries. The carotid arteries were isolated from the carotid sheath and vagus nerve, and carotid arteries were ligated with a 5/0 silk suture. Rats in the sham group were operated in the same surgical procedure except that the arteries were not ligated.

### 2.4. Behavioral Analysis

The autonomic motor of rats was assessed by OFT, and spatial memory capability of rats was assessed by MWM test as previously reported [18, 19]. The rats were transferred to the behavioral test room (soundproof dark room) before behavioral test. OFT was performed from 19:00 to 24:00. The open field is a black box (100 × 100 × 40 cm), and for data analysis, the total area was equally divided into 25 equal squares virtually. Each rat was gently placed in the center of the field and observed for 5 min. Between trials, the floor of the box was wiped with 50% ethanol to remove the scent marks. The total distance (mm) and average velocity (mm/s) were recorded and analyzed. MWM was performed in the experimental apparatus consisted of a circular tank (150 cm in diameter and 50 cm in depth) divided into four quadrants and filled with water dyed with black food colorant (24 ± 1°C). A circular platform (12 cm in diameter and 30 cm in depth) was submerged about 1 cm below the water surface in the south-west quadrant. Space navigation test was conducted during days 1-5 with 4 trials per day, and one trial each day was from each of the four positions selected randomly. The interval between trials was 10 min. The rats were given 60 s to locate the hidden platform and allowed to rest on the platform for 10 s. The space exploration test was allowed to swim freely for 60 s. Escape latency (s) in the space navigation test, number of crossing platform, the time during platform quadrant (s), and average speed (mm/s) in space exploration test were recorded and analyzed. All behavioral results were analyzed by video-tracking system (Flydy Co., Ltd., Guangzhou, China).

### 2.5. Histological Evaluation

The animal used for histological evaluation were perfused, firstly with 0.9% saline and then with cold 10% paraformaldehyde. The brains were removed, washed, and protected in the 10% paraformaldehyde. Formalin-fixed brains were dehydrated in a series of graded ethanol and xylene baths and embedded in paraffin. Brains were cut into 6 *μ*M thick coronal sections using a RM2255 microtome (Leica, Switzerland), mounted on glass slides. Distribution of neural cells in the rat prefrontal cortex and striatum was assessed by microscopy following standard hematoxylin-eosin (HE) staining (Servicebio, China). Nissl bodies in rat brain tissues were detected by a previously described Nissl staining method (Solarbio, China) [18].

### 2.6. Immunofluorescence

After dewaxing and rehydrating and antigen retrieval, the paraffin sections were blocked endogenous peroxidase activity by citric buffer (Pondus Hydrogenii (PH) 6.0). Brain sections were blocked with normal goat serum for 1 h at room temperature. The primary antibody was diluted in phosphate buffer saline (PBS) (PH 7.4) in a certain proportion (tyrosine hydroxylase (TH), Proteintech, 1 : 200) and is added to the sections, and the sections are placed flat in a wet box and incubated overnight at 4°C. Following three washes with PBS, rat brain sections were incubated in the dark with green fluorophore-conjugated secondary antibodies for 1.5 h at room temperature. After counterstaining with 4′,6-diamidino-2-phenylindole dihydrochloride (DAPI), sections were mounted and observed by fluorescence microscopy.

### 2.7. Immunocytochemistry

The steps for dewaxing and rehydrating, antigen retrieval, blocking endogenous peroxidase activity, and serum sealing are similar to immunofluorescence. The primary antibody was diluted in PBS (PH 7.4) in a certain proportion (*α*-Syn, Abcam, 1 : 16000; A*β*, Abcam, 1 : 200) and is added to the sections, and the sections are placed flat in a wet box and incubated overnight at 4°C. After the sections are slightly shaken and dried, the tissues are covered with secondary antibody (horseradish peroxidase labeled) from the corresponding species of primary antibody and incubated at room temperature for 50 minutes. 3,3-N-diaminobenzidine tertrahydrochloride (DAB) color developing solution newly prepared is added in the circle after the sections are slightly dried. After counterstaining nucleus, the brain sections were dehydrated and mounted. Then, sections were observed by microscopy.

### 2.8. RNA Sequencing and Bioinformatics

Differential expressions of messenger RNA (mRNA) in prefrontal cortex of the sham group and PD+BCCAO 2 weeks were characterized by RNA sequencing (RNA-Seq). Total RNA was prepared from prefrontal cortex using TRIzol reagent (Thermo Fisher Scientific, USA) according to the manufacturer's instructions. The concentrations of RNA were assessed by NanoDrop 2000 spectrophotometer (Thermo Scientific, USA), and the quality of RNA was analyzed calculating RNA integrity number with Agilent Bioanalyzer 2100 (Agilent Technologies). Transcriptome sequencing was performed on RNA samples that passed the quality control indicators (2100 RNA integrity number (RIN) ≥ 7.0 and 28S/18S > 1.0). Subsequently, the RNA-Seq libraries were constructed and sequenced on the DNBSEQ platform (Beijing Genomics Institution, China). In order to display the number of genes in different expression intervals of each sample more intuitively, we carried out statistics on the number of genes in three cases of expression amount (expression ≤ 1, expression = 1 − 10, and expression ≥ 10). Following adapter trimming and other processing, we used HISAT (http://ccb.jhu.edu/software/hisat/index.shtml) and Bowtie2 (http://bowtie-bio.sourceforge.net/bowtie2/index.shtml) to align clean reads to the reference genome sequence (NCBI, GCF_000001895.5_Rnor_6.0) to get the alignment results. Differential expressions of mRNAs between the two groups were defined as the combination of a log2 Ratio (PD+BCCAO 2 weeks/PD) value of ≥1 and a *P* value ≤ 0.05 based on reads per kilobase million using DESeq2 software. Hierarchical clustering and scatter plots of differentially expressed mRNAs were constructed using R language. Enrichment of Gene Ontology (GO) and Kyoto Encyclopedia of Genes and Genomes (KEGG) pathways for differentiation expressed mRNAs was performed using the Database for Annotation, Visualization, and Integrated Discovery system (DAVID, https://david.ncifcrf.gov/). The GO and KEGG pathways with *P* values (*P*) < 0.05 were considered significant.

### 2.9. Sample Preparation and Ultraperformance Liquid Chromatography-High-Definition Mass Spectrometry (UPLC-HDMS) Analysis

The collected samples in the prefrontal cortex were thawed on ice, and metabolites were extracted with 50% methanol buffer. Pooled quality control (QC) samples were also prepared by combining 10 *μ*L of each extraction mixture. All samples were acquired by the LC-MS system following machine orders. Firstly, all chromatographic separations were performed using a Thermo Scientific UltiMate 3000 HPLC (Thermo Scientific, USA). An ACQUITY UPLC BEH C18 column (100 mm∗2.1 mm, 1.8 *μ*m, Waters, UK) was used for the reversed phase separation. The column oven was maintained at 35°C. The flow rate was 0.4 mL/min, and the mobile phase consisted of solvent A (water, 0.1% formic acid) and solvent B (acetonitrile, 0.1% formic acid). Gradient elution conditions were set as follows: 0~0.5 min, 5% B; 0.5~7 min, 5% to 100% B; 7~8 min, 100% B; 8~8.1 min, 100% to 5% B; and 8.1~10 min, 5%B. The injection volume for each sample was 4 *μ*L. A high-resolution tandem mass spectrometer Q-Exactive (Thermo Scientific, USA) was used to detect metabolites eluted from the column. The Q-Exactive was operated in both positive and negative ion modes. Precursor spectra (70–1050 m/z) were collected at 70,000 resolution to hit an AGC target of 3e6. The maximum inject time was set to 100 ms. A top 3 configuration to acquire data was set in DDA mode. Fragment spectra were collected at 17,500 resolution to hit an AGC target of 1e5 with a maximum inject time of 80 ms. In order to evaluate the stability of the LC-MS during the whole acquisition, a quality control sample (pool of all samples) was acquired after every 10 samples. MetaX software was used to quantify and screen the differential metabolites. Those features that were detected in less than 50% of QC samples or 80% of biological samples were removed, the remaining peaks with missing values were imputed with the k-nearest neighbor algorithm to further improve the data quality. PCA was performed for outlier detection and batch effect evaluation using the preprocessed dataset. Quality control-based robust LOESS signal correction was fitted to the QC data with respect to the order of injection to minimize signal intensity drift over time. In addition, the relative standard deviations of the metabolic features were calculated across all QC samples, and those >30% were then removed.

### 2.10. Pattern Recognition Analysis and Data Processing

The raw data of mass spectrometry was converted into readable data mzXML using MSConvert software. Peak extraction was performed using XCMS software, and peak extraction quality control was performed. The extracted substances were annotated by summation ions using CAMERA and then identified by MetaX software. The MS primary information was used for identification, and the MS secondary information was matched to an in-house standard database, respectively. Metabolite annotation was performed on candidate identification substances using databases such as HMDB (https://hmdb.ca/) and KEGG, to explain the physicochemical properties and biological functions of metabolites. Student's *t*-tests were conducted to detect differences in metabolite concentrations between 2 groups. The *P* value was adjusted for multiple tests using an false discovery rate (Benjamini–Hochberg). Supervised PLS-DA was conducted through MetaX to discriminate the different variables between groups. The variable important in the projection (VIP) value was calculated. Differential metabolic ions between the two groups were defined as the combination of a ratio ≥ 1.5 or ≤1/1.5, a *P* value ≤0.05, and VIP ≥ 1. Enrichment of KEGG pathways for differentiation lipids was performed using the DAVID.

### 2.11. Quantitative Real-Time Polymerase Chain Reaction

Total RNA samples were extracted from prefrontal cortex using TRIzol according the manufacturer's instructions. Concentrations and quality of total RNA samples were measured using a NanoDrop 2000 spectrophotometer. Complementary deoxyribonucleic acid (cDNA) was synthesized from 500 ng of RNA from each group by reverse transcription using a RevertAid First Strand cDNA Synthesis Kit (Thermo Scientific, USA). In CFX connect Real-Time System (Bio-Rad, USA), a GoTaq qPCR Master Mix kit (Promega, USA) was used for quantitate expression levels by quantitative PCR. Relative expressions of mRNAs were calculated using the standard 2^-∆∆Ct^ method for at least three biological replicates [18]. Also, *β*-actin levels are used for calibration. Primer sequences are listed in Table 1.

### 2.12. Statistical Analysis

Statistical tests were carried out using SPSS 26.0 software. Mauchly's test of sphericity was used to analyze repeated measurement data. Data considered to conform to a normal distribution (*P* value of the Shapiro-Wilk test > 0.05) were presented as the mean ± standard deviation and analyzed by one-way analysis of variance (ANOVA). Data that did not conform to a normal distribution were presented as median (mix-max) and analyzed by nonparametric tests. *P* < 0.05 was considered significant. GraphPad Prism 6.0 software was used to draw images.

## 3. Results

### 3.1. CCH Aggravated PDD-Like Symptoms in 6-OHDA-Lesioned Rat

**Figure 2:**
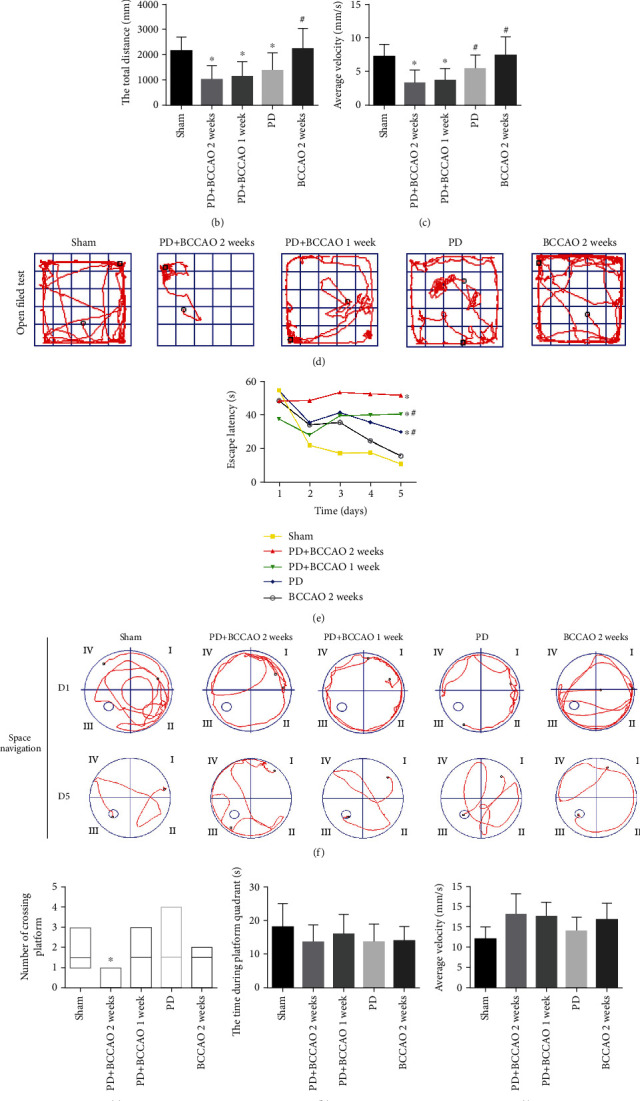
Behavioral performance in apomorphine-induced rotation tests, OFT, and MWM (*n* = 10). (a) The number of rotations of each group in apomorphine-induced rotation tests. (b) The total distance of each group in OFT test. (c) Average velocity of each group in OFT test. (d) Representative movement trajectories of rats in the OFT. (e) Escape latency of each group in space navigation of MWM. (f) Representative movement trajectories of rats on the first and fifth days of space navigation. (g) Number of crossing platform of each group in space exploration of MWM. (h) The time during platform quadrant of each group in space exploration of MWM. (i) Average speed of each group in space exploration of MWM. (j) Representative movement trajectories of rats in space exploration of MWM. ^∗^*P* < 0.05 compared with the sham group and ^#^*P* < 0.05 compared to the BCCAO 2 weeks group.

To validate the effect of CCH on the behavior of 6-OHDA-lesioned rats, we assessed the motor and cognitive functions of the rats in each group by apomorphine-induced rotation, OFT, and MWM (Figure 2). Firstly, apomorphine-induced rotation tests were used to characterize the dopamine depletion levels after unilateral 6-OHDA infusion in rats. The 6-OHDA-lesioned rats with contralateral rotations were divided into three groups: PD+BCCAO 2 weeks group, PD+BCCAO 1 week group, and PD group for the next step of the experiment (supplementary Figure 1A). Then, compared with PD group, we found that CCH could not aggravate the number of rotations induced by apomorphine in PD+BCCAO 2 weeks group and PD+BCCAO 1 week group (*P* > 0.05, Figure 2(a)). Interestingly, we found that there was no significant difference in the body weight of the rats in each group during the experimental period (supplementary Figure 1B).

In addition, compared with the sham group, OFT results demonstrated a significant decrease in total distance and average velocity traveled by rats in the PD group, PD+BCCAO 2 weeks group, and PD+BCCAO 1 week group (*P* < 0.05, Figures 2(b)–2(d)). Interestingly, compared with the PD group, CCH caused the animal to exhibit a slower average velocity (*P* < 0.05, Figure 2(c)). The above results indicated that CCH could aggravate PDD-like motor symptom, especially voluntary motor velocity.

Cognitive functions, including learning-memory and spatial cognition were evaluated using the MWM [20]. In the space navigation tests, PD+BCCAO 2 weeks group showed a significantly increased in the time of escape latency, compared to the sham group, PD group, and PD+BCCAO 1 week group (*P* < 0.05, Figures 2(e) and 2(f)). Also, compared to the sham group, PD+BCCAO 2 weeks group showed a significantly decreased in the number of crossing platform in the space exploration test (*P* < 0.05, Figures 2(g) and 2(j)). But the time during platform quadrants and average speed in the space exploration test, there was no significant difference in the results of each group (*P* > 0.05, Figures 2(h)–2(j)). It manifested that the number of crossing platform in the space exploration test was not affected by the swimming speed of the animal in the water. In addition, the time of escape latency and the number of crossing platform reflect learning memory and spatial cognition, respectively. Thus, these results indicated that CCH could aggravate PDD-like cognitive symptoms, including learning memory and spatial cognition, especially learning memory.

### 3.2. CCH Damaged the Structure and Neuronal Cells in Prefrontal Cortex and Striatum of 6-OHDA-Lesioned Rat

**Figure 3:**
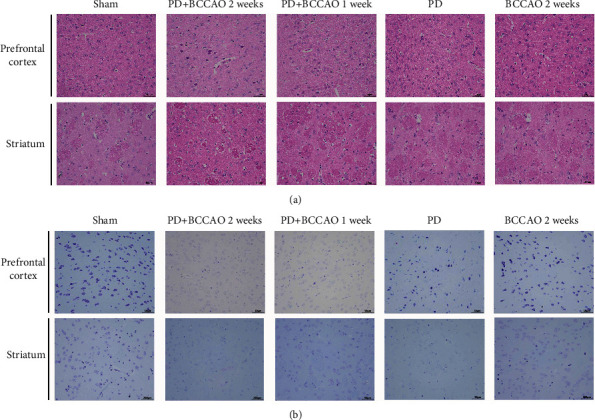
Hematoxylin and eosin (H&E) staining and Nissl staining sections from prefrontal cortex and striatum (*n* = 3). (a) Representative images of HE staining in prefrontal cortex and striatum of each group. In PD+BCCAO 2 weeks group, the interstitial space of the striatum is relatively loose, while the boundary of the striatal interstitial space is unclear, and many vacuoles can be seen throughout the striatum. The prefrontal cortex in PD+BCCAO 2 weeks group showed that a large number of neurons in the prefrontal cortex were shrunken, the staining of the cells was deepened, the boundary between the nucleus and cytoplasm was unclear, and the cells were loosely arranged. (b) Representative images of Nissl staining in prefrontal cortex and striatum of each group. Dark blue represents Nissl bodies. Nissl bodies are large and numerous, indicating that nerve cells have a strong function of synthesizing proteins; on the contrary, when nerve cells are damaged, the number of Nissl bodies will decrease or even disappear. The numbers of Nissl-stained neurons in prefrontal cortex and striatum were significantly decreased in the PD+BCCAO 2 weeks group. Scale bars represent 50 *μ*m.

Previous literature reported that learning memory is related to prefrontal cortex and spatial cognition is related to striatum [21–24]. To explore the effect of CCH on brain tissue, we further analyzed the morphological structures of prefrontal cortex and striatum. In PD+BCCAO 2 weeks group, the interstitial space of the striatum is relatively loose, while the boundary of the striatal interstitial space is unclear, and many vacuoles can be seen throughout the striatum (Figure 3(a)). Also, compared with the others groups, the prefrontal cortex in PD+BCCAO 2 weeks group showed that a large number of neurons in the prefrontal cortex were shrunken, the staining of the cells was deepened, the boundary between the nucleus and cytoplasm was unclear, and the cells were loosely arranged (Figure 3(a)). In addition, numbers of Nissl-stained neurons in prefrontal cortex and striatum were significantly decreased in the PD+BCCAO 2 weeks group compared with the other groups (Figure 3(b)).

### 3.3. CCH Aggravated PDD-Like Pathology in Prefrontal Cortex and Striatum of 6-OHDA-Lesioned Rat

**Figure 4:**
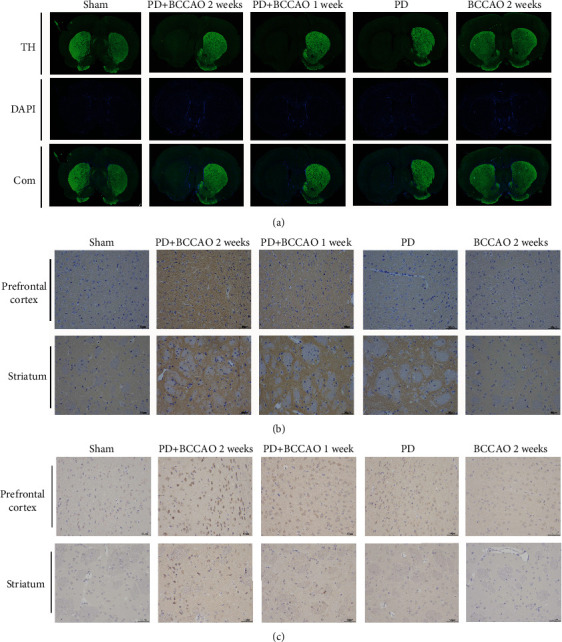
CCH aggravated PDD-like pathology in prefrontal cortex and striatum of 6-OHDA-lesioned rat (*n* = 3). (a) Representative images of TH expression in prefrontal cortex and striatum of each group. TH (green) and DAPI nuclear stain (blue). CCH could not aggravate the loss of TH-positive cells in striatum, compared to the PD group. (b) Representative images of *α*-Syn expression in prefrontal cortex and striatum of each group. *α*-Syn (brown) and cell nucleus (blue). CCH combined with 6-OHDA treatment could increase the expression of *α*-Syn in prefrontal cortex. (c) Representative images of A*β* expression in prefrontal cortex and striatum of each group. A*β* (brown) and cell nucleus (blue). The expression of A*β* in striatum and prefrontal cortex of PD+BCCAO 2 weeks was higher than that of other groups. Scale bars represent 50 *μ*m.

TH is a critical marker of dopaminergic neurons. Therefore, we explored the effect of CCH on dopaminergic neurons of striatum. As shown in Figure 4(a), compared with the sham group, 6-OHDA treatment led to decrease of TH-positive cells in the 6-OHDA-lesioned striatum. However, CCH could not aggravate the loss of TH-positive cells in striatum, compared to PD group.

In addition, *α*-Syn and A*β* are the major pathological markers of PDD. We found that 6-OHDA treatment would increase the expression of *α*-Syn in striatum, compared with the sham group and BCCAO 2 weeks group (Figure 4(b)). Also, compared with the sham group, PD group, and BCCAO 2 weeks group, CCH combined with 6-OHDA treatment could increase the expression of *α*-Syn in prefrontal cortex (Figure 4(b)). Moreover, the expression of A*β* in striatum and prefrontal cortex of PD+BCCAO 2 weeks was higher than that of other groups (Figure 4(c)). The above results indicated that CCH combined with 6-OHDA treatment aggravated PDD-like pathology in prefrontal cortex and striatum, especially prefrontal cortex. Simultaneously, the pathology of PD+BCCAO 2 weeks group was more obvious than PD+BCCAO 1 week. Therefore, to further explore the mechanism by which CCH causes PDD-like pathology, we selected prefrontal cortex form PD group and PD+BCCAO 2 weeks group for RNA-Seq.

### 3.4. CCH Modulated mRNA Expression in Prefrontal Cortex of 6-OHDA-Lesioned Rat

**Figure 5:**
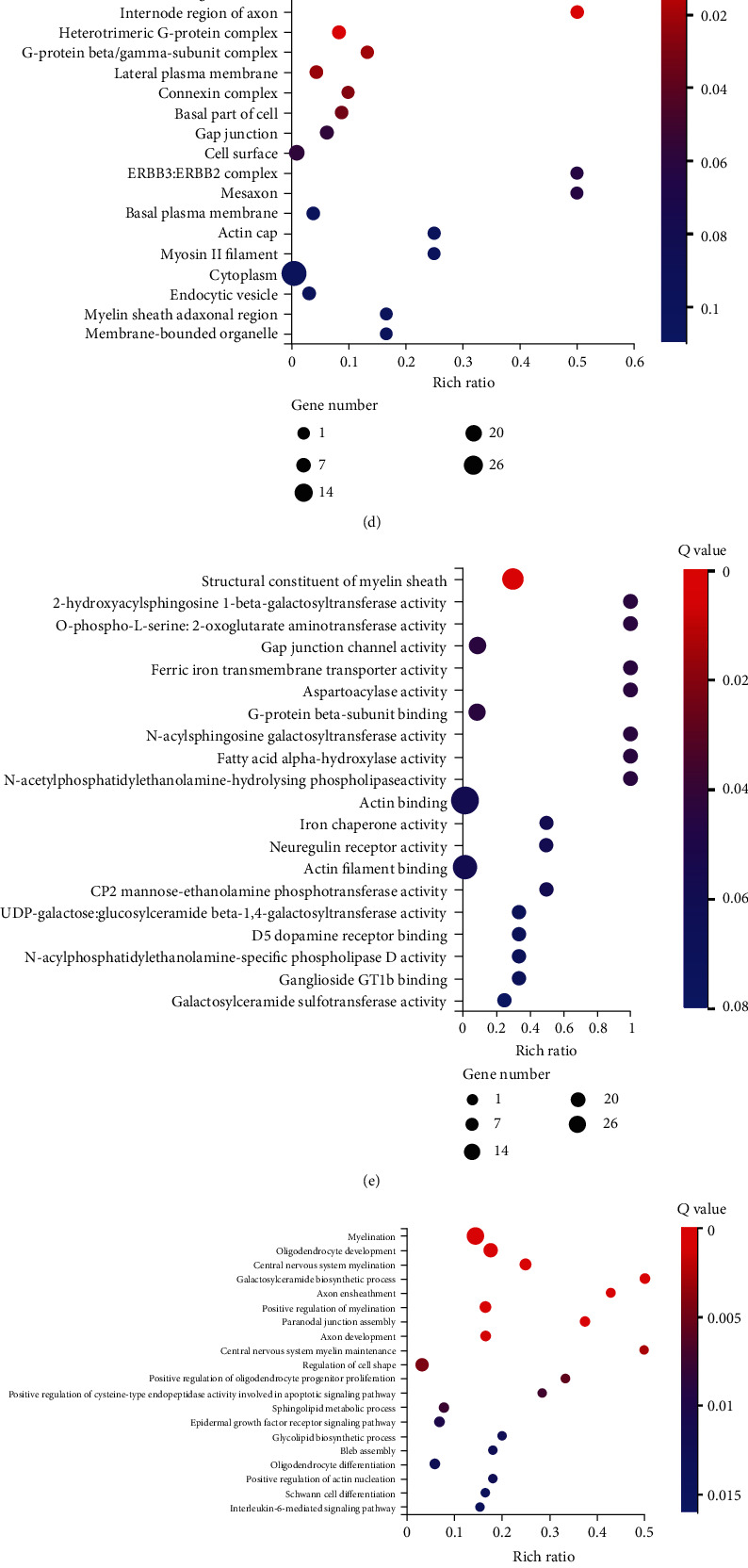
CCH modulated mRNA expression in prefrontal cortex of 6-OHDA-lesioned rat (*n* = 3). (a) Volcano map of differentially expressed mRNAs characterized by RNA sequencing. Compared with the PD group, green represents decrease in the PD+BCCAO 2 weeks group, and red represents increase. (b) Correlation heatmap of differentially expressed mRNAs. The color represents the expression level; the higher the expression level, the redder the color, otherwise the bluer. (c) PPI of differentially expressed mRNAs. (d) Enrichment of GO biological process of differentially expressed mRNAs. (e) Enrichment of GO cellular component of differentially expressed mRNAs. (f) Enrichment of GO molecular function of differentially expressed mRNAs. (g) Enrichment of KEGG pathways of differentially expressed mRNAs.

Overall, 61 differentially expressed mRNAs, including 11 upregulated and 50 downregulated mRNAs, were identified in the PD+BCCAO 2 weeks group compared with the PD group (Figures 5(a)–5(c)). Also, protein-protein interaction network (PPI) showed that Mag, Mbp, Plp1, Mobp, and Fa2h have more associations with other mRNAs (Figure 5(c)). Enrichment of GO cellular component showed that the differentially expressed mRNAs involve in myelin sheath, compact myelin, and paranode region of axon (Figure 5(d)). Enrichment of GO molecular function showed that the differential mRNAs possess molecular functions including structural constituent of myelin sheath, fatty acid alpha-hydroxylase activity, and N-acetylphosphatidylethanolamine-hydrolysing phospholipase activity (Figure 5(e)). Moreover, differentially expressed mRNAs are involved in biological processes, including myelination, galactosylceramide biosynthetic process and sphingolipid metabolic process (Figure 5(f)). Then, KEGG analysis demonstrated that differentially expressed mRNAs related to CCH were significantly enriched in multiple signaling pathways, including sphingolipid signaling pathway, sphingolipid metabolism, phospholipase D signaling pathway, and ether lipid metabolism (Figure 5(g)). It is clear that there is a potential relationship between differentially expressed mRNAs and lipid metabolism. In order to elucidate the metabolism of which lipids are specifically affected by CCH, we performed lipidomics on prefrontal cortex.

### 3.5. CCH Aggravates PDD-Like Symptoms and Pathology in 6-OHDA-Lesioned Rat through Interfering with Sphingolipid Metabolism

**Figure 6:**
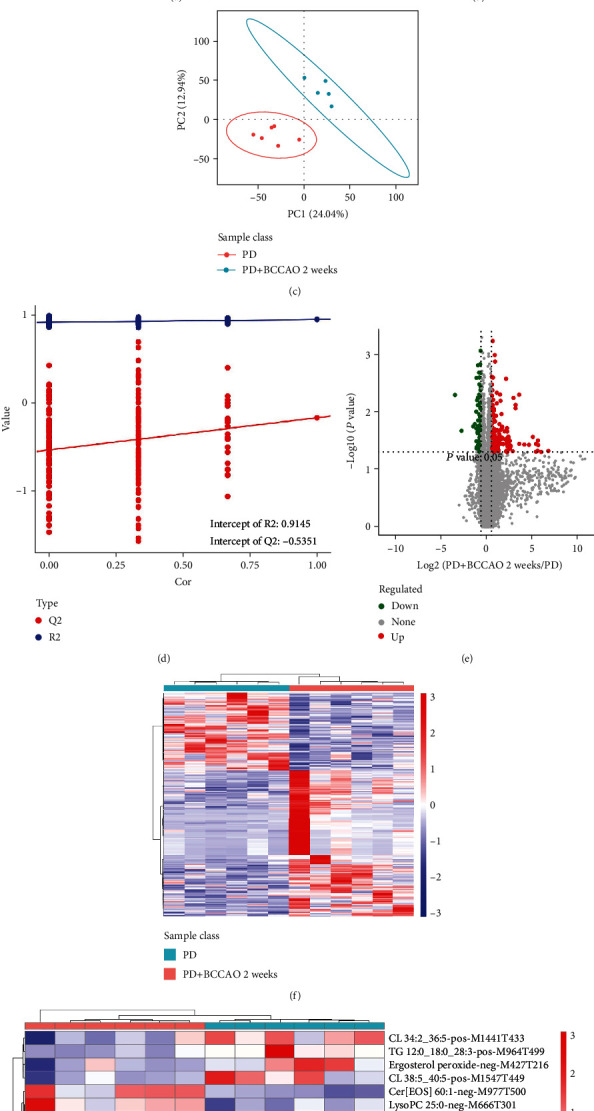
CCH aggravates PDD-like symptoms and pathology in 6-OHDA-lesioned rat through interfering with sphingolipid metabolism (*n* = 6). (a) Metabolic ions identified in positive and negative ion mode into six major categories of lipids. (b) PCA analysis of differentially metabolic ions. The horizontal axis is the first principal component, and the vertical axis is the second principal component. (c) PLS-DA analysis of differentially metabolic ions. (d) Response ranking test plot for the PLS-DA analytical model. (e) Volcano map of differentially metabolic ions. (f) Correlation heatmap of differentially metabolic ions. (g) Correlation heatmap of differentially secondary metabolites. (h) Enrichment of KEGG pathways of differentially metabolic ions.

The QC information of samples was shown in supplemental Figure 2. As shown in Figure 6(a), we have classified the 7069 metabolic ions identified by negative ion mode and 13970 metabolic ions identified in positive ion mode into six major categories of lipids including fatty acyls (FA), glycerolipids (GL), glycerophospholipids (GP), prenol lipids (PR), sphingolipids (SP), and sterol lipids (ST). Then, differential lipids in the prefrontal cortex of the PD group and the PD+BCCAO 2 weeks group were screened using multivariate statistical analysis methods principal component analysis (PCA) (PC1 = 28.92%, PC2 = 16.53%, PC3 = 11.39%) and partial least squares method-discriminant analysis (PLS-DA) (*R*2 = 0.946889, *Q*2 = −0.1709, *P* value-*R*2 = 0.195, *P* value-*Q*2 = 0.245, intercept-*R*2 = 0.91, intercept-*R*2 = −0.54) (Figures 6(b)–6(d)). The results suggested that the main metabolic ions of this study can be analyzed by the above two models.

Differential metabolic ions between the two groups were defined as the combination of a ratio ≥ 1.5 or ≤1/1.5, a *P* ≤ 0.05, and VIP ≥ 1. Overall, 273 differential metabolic ions, including 178 upregulated and 95 downregulated metabolic ions, were identified in the PD+BCCAO 2 weeks group compared with the PD group (Figures 6(e) and 6(f)). Among, there are 18 metabolic ions for which secondary metabolites can be identified, including pos-M716T311 (SM 9:0;2O/26:2), pos-M690T309 (SM 8:1;2O/25:0), pos-M726T325 (SM 8:0;2O/28:4), neg-M711T417 (Cer[BDS 42:1]), neg-M697T409 (Cer[BS]), neg-M977T500 (Cer[EOS]), etc. (Figure 6(g)). Finally, compared with the PD group, the content of 5 kinds of lipids, including sphingomyelin, cardiolipin, lysophosphatidylcholine, cholesteryl ester, and triacylglycerol, significantly changed in the PD+BCCAO 2 weeks group. Interestingly, enrichment of KEGG signaling pathway of 5 kinds of lipids also suggested that CCH mainly affected the sphingolipid signaling pathway and sphingolipid metabolism (Figure 6(h)).

**Figure 7:**
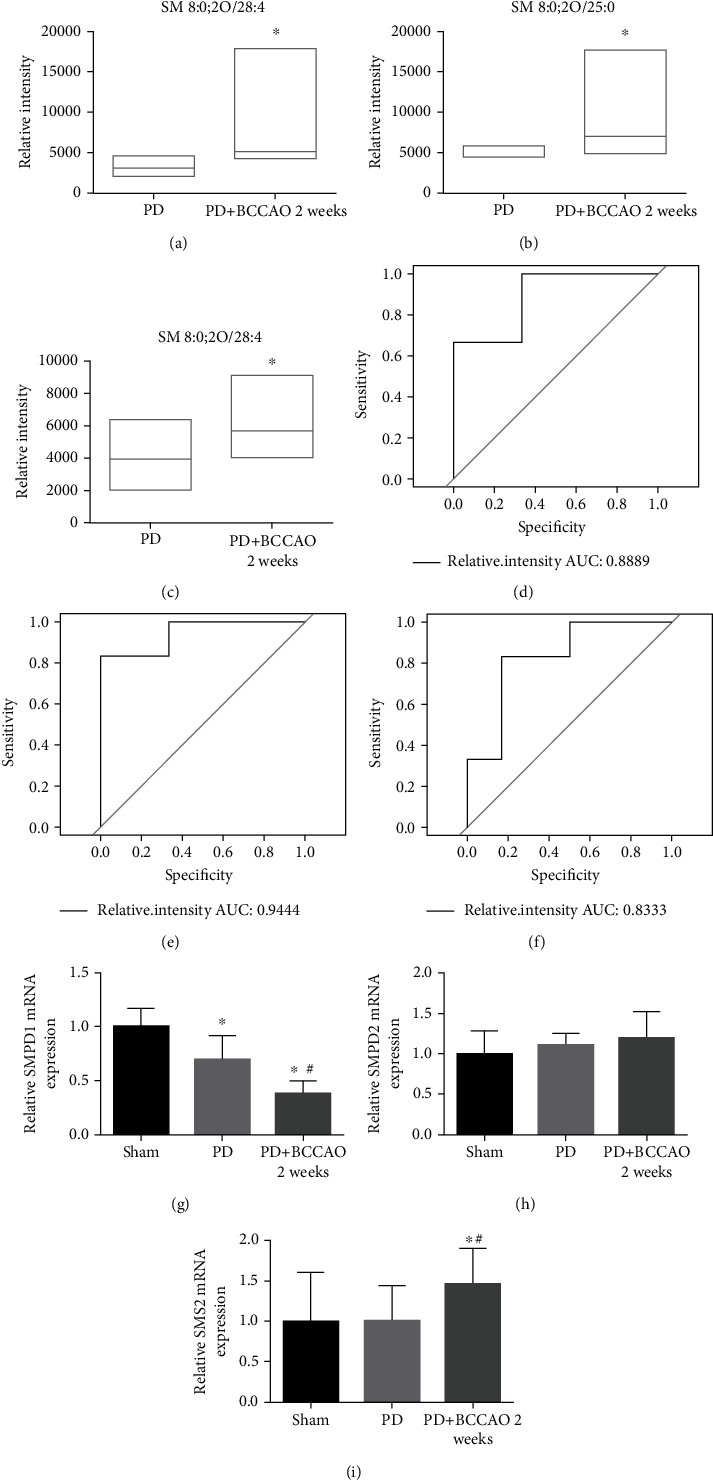
CCH disrupts the sphingolipid metabolism by affecting the mRNA expression of SMPD1 and SMS2. (a–c) Relative intensity analysis of metabolic ions. pos-M716T311 (SM 9:0;2O/26:2), pos-M690T309 (SM 8:1;2O/25:0), and pos-M726T325 (SM 8:0;2O/28:4) are higher in the PD+BCCAO 2 weeks group compared to the PD group. (d) Receiver operating characteristic curve analysis showed the AUC of pos-M726T325 (SM 8:0;2O/28:4) was 0.8889. (e) Receiver operating characteristic curve analysis showed the AUC of pos-M690T309 (SM 8:1;2O/25:0) was 0.9444. (e) Receiver operating characteristic curve analysis showed the AUC of pos-M716T311 (SM 9:0;2O/26:2) was 0.8333. (g–i) Relative mRNA expression of each group. The relative expression of SMPD1 was significantly decreased in the prefrontal cortex of PD+BCCAO 2 weeks group, but SMS2 was significantly elevated, while SMPD2 did not change. ^∗^*P* < 0.05 compared with the sham group and ^#^*P* < 0.05 compared to BCCAO 2 weeks group.

Since the lipid involved in the sphingolipid metabolism is sphingomyelin, we further showed the relative intensity of metabolic ions, including pos-M716T311, pos-M690T309, pos-M726T325 (Figures 7(a)–7(c)). To further identify biomarkers, the screened differential metabolic ions were analyzed using partial least squares-based ROC curves. We found that the area under the curve (AUC) of pos-M716T311, pos-M690T309, and pos-M726T325 is more than 0.8. Also, sensitivity and specificity of above metabolic ions are more than 80% (Figures 7(d)–7(f)). These results suggested that sphingomyelin may serve as a potential biomarker for a PDD-like model induced by CCH combined with 6-OHDA treatment.

In order to explore the abnormal increase in the level of Sphingomyelin, we detected the mRNA associated with the synthesis (SMPD1 and SMPD2) and decomposition (SMS2) of sphingomyelin. Compared with the sham group and PD group, we found the relative expression of SMPD1 was significantly decreased in the prefrontal cortex of PD+BCCAO 2 weeks group, but SMS2 was significantly elevated, while SMPD2 did not change (Figures 7(g)–7(i)). These results suggested that CCH disrupts the sphingolipid metabolism by affecting the mRNA expression of SMPD1 and SMS2, leading to the accumulation of sphingomyelin in the prefrontal cortex.

## 4. Discussion

According to the diagnosis and treatment guidelines, the two important symptoms of PDD are motor deficits (i.e., bradykinesia, postural instability, asymmetric resting, and rigidity) and cognitive deficits in memory, attention, executive function, and visuoconstructive ability [25]. Meanwhile, CCH is a cardinal risk factor for PDD, especially for cognitive impairment [7, 26–29]. To elucidate the mechanism of CCH exacerbating cognitive impairment in PD, we used BCCAO to simulate CCH in rats with reference to previous literature [30, 31]. Importantly, our results also certified that CCH could aggravate PDD-like symptoms in 6-OHDA-lesioned rats including bradykinesia (Figures 2(b)–2(d)) and memory impairment (Figures 2(e)–2(j)). Usually, WMW is mainly used to assess learning memory impairment and spatial orientation memory impairment in animals. As shown in Figure 2(f), during the learning process of spatial navigation, the PD+BCCAO 2 weeks group still showed marginal movement after 4 days of learning. Also, the PD+BCCAO 2 weeks group showed marginal movement and spatial memory impairment in the recall of spatial exploration (Figure 2(j)). These results suggested that CCH could seriously affect the learning ability of animals, resulting in animals unable to remember the location of the platform.

Then, we found that decreased neuronal cells (Figure 3) and increased expression of *α*-Syn and A*β* (Figures 4(b) and 4(c)) in the prefrontal cortex and striatum. The most persuasive evidence to date suggests that *α*-Syn pathology and A*β* pathology, which are widely distributed in brain of PDD patients, plays a paramount role in PDD [32–34]. Therefore, CCH can aggravate not only PDD-like symptoms but also PDD-like pathology in 6-OHDA-lesioned rat. Also, the complete circle of Willis in the rat affords incessant (but reduced) blood flow from the vertebral arteries after the onset of occlusion. The combination of BCCAO and 6-OHDA-lesioned can better simulate the disease state of PDD.

Prefrontal cortex is closely related to higher cognitive functions such as attention, memory, and learning. Also, ipsilateral nigrostriatal dopaminergic function was related to prefrontal cortex perfusion [35]. Coincidently, the PD+BCCAO 2 weeks group performed serious learning impairment in MWM, which is related to prefrontal cortex (Figure 2). Meantime, PDD-like pathology could be found in prefrontal cortex (Figure 4). Thus, we speculated that prefrontal cortex is an important lesion location in relation to cognitive impairment. So, we further explore the mechanism of effect of CHH on prefrontal cortex by RNA-Seq and lipidomics.

According to the analysis of RNA-Seq and lipidomics, we found that CCH mainly disrupts the sphingolipid metabolism by affecting the mRNA expression of SMPD1 and SMS2, leading to the accumulation of sphingomyelin in the prefrontal cortex (Figures 5–7). Sphingolipids are a group of structurally diverse lipids which play an integral role in body functions, including participation in cell proliferation, death, migration, membrane domains and signaling, and central nervous system development [36]. Sphingolipids mainly include sphingosine, sphingomyelin, sphingolipid intermediates, ceramides, and sphingosine-1-phosphate. Sphingomyelin is a complex sphingolipid, consisting of phosphorylcholine and ceramide, in a cylindrical structure, which is responsible for myelination, impulse transmission, synaptic plasticity, localization of neurotransmitter receptors, and blood circulation. Integrity of the brain barrier is critical [37, 38]. Elevated levels of SM (C18:1) and SM (C20:0) in the anterior cingulate cortex of PD patients, which is similar to our findings [39, 40].

The catabolism and synthesis of sphingomyelin are mediated by sphingomyelinase (SMases, encoded by SMPD1 and SMPD2) and sphingomyelin synthase 2 (SMS2, encoded by SMS2), respectively. Thus, high-level of sphingomyelin might be related to SMPD1, SMPD2, and SMS2. Our results showed that mRNAs of low-expressed SMPD1 and high-expressed SMS2, leading to accumulation of sphingomyelin in the prefrontal cortex (Figure 7). On the one hand, abnormally elevated sphingomyelin promotes the accumulation of *α*-Syn in the plasma membrane region, which disrupts plasma membrane organization and leads to the degradation of synaptic function [41, 42]. On the other hand, sphingomyelin could promote the expression of *α*-Syn and hinder the decomposition of *α*-Syn [43]. Mutation of the gene SMPD1 encoding lysosomal acid SMase can lead to abnormal lysosomal function and affect the degradation of *α*-Syn in lysosomes [44, 45]. Therefore, we speculated that CCH can aggravate the aggregation and expression of *α*-Syn, which is related to cognitive functions, by promoting the accumulation of sphingomyelin in the prefrontal cortex.

## 5. Conclusion

In present study, the effects and mechanisms of CCH on PDD-like symptoms and pathology were evaluated in the classical PD rat model of 6-OHDA-lesions. In summary, we found that CCH is an independent exacerbating reason for impairment in learning and memory within the pathopoiesis of PD. Also, CCH disrupts the sphingolipid metabolism by affecting the mRNA expression of SMPD1 and SMS2, leading to the accumulation of sphingomyelin in the prefrontal cortex. Elevated levels of sphingomyelin could aggravate the aggregation and expression of *α*-Syn, which is related to cognitive functions. Thus, maintenance of adequate cerebral perfusion and stable sphingolipid metabolism may be critical to prevent further cognitive impairment in PD patients.

## Figures and Tables

**Table 1:** A list of primers used for qRT-PCR assay.

Primer ID	Primer sequences (5′-3′)
*β*-Actin-F	AAGATCCTGACCGAGCGTGG
*β*-Actin-R	CACAGGATTCCATACCCAGGAAG
SMPD1-F	ATGAGGAAACTCTGAGCCGC
SMPD1-R	GGTAAACTCGGTAGCCAGGA
SMPD2-F	CCTACGTGACCCATCTGCAC
SMPD2-R	TCCTCGGTCTCAACAAAGGC
SMS2-F	CAGTGTGCTCCAAAGCTCAA
SMS2-R	TCGACCGTGTAGTGTTCGTG

## Data Availability

The datasets generated and/or analysed during the current study are not publicly available because the data still need further analysis, but are available from the corresponding author on reasonable request.

## Supplementary Materials

Supplementary Materials Supplemental Figure 1: (A) the number of rotations of each group in apomorphine-induced rotation tests. (B) Weight monitoring during the experiment. Supplemental Figure 2: the QC information of samples. (A) Negative ion mode TIC overlay of QC samples. (B) Metabolic ions distribution in negative ion mode. (C) Positive ion mode TIC overlay of QC samples. (D) Metabolic ions distribution in positive ion mode. (E) PCA analysis of all identified metabolic ions. (F) Correlation analysis of all identified metabolic ions.
