# Correction to “Chronic Cerebral Hypoperfusion Aggravates Parkinson’s Disease Dementia‐Like Symptoms and Pathology in 6‐OHDA‐Lesioned Rat through Interfering with Sphingolipid Metabolism”

**DOI:** 10.1155/omcl/9801758

**Published:** 2026-04-05

Y. Fan, M. Li, C. Wu, et al., “Chronic Cerebral Hypoperfusion Aggravates Parkinson’s Disease Dementia‐Like Symptoms and Pathology in 6‐OHDA‐Lesioned Rat through Interfering with Sphingolipid Metabolism,” *Oxidative Medicine and Cellular Longevity*, vol. 2022 (2022). https://doi.org/10.1155/2022/5392966.

In the article titled “Chronic Cerebral Hypoperfusion Aggravates Parkinson’s Disease Dementia‐Like Symptoms and Pathology in 6‐OHDA‐Lesioned Rat through Interfering with Sphingolipid Metabolism,” there was an error in Figure 3a.

More specifically, the TH staining image of the striatum in the PD group was mistakenly duplicated with the BCCAO 2  weeks  group’s image. Re‐verification confirmed the BCCAO 2  weeks  group’s striatal TH staining was incorrect. This error occurred during figure assembly and the corrected figure is shown below, and listed as Figure 3:

Figure 3Hematoxylin and eosin (H&E) staining and Nissl staining sections from prefrontal cortex and striatum (*n* = 3). (a) Representative images of HE staining in prefrontal cortex and striatum of each group. In PD + BCCAO 2 weeks group, the interstitial space of the striatum is relatively loose, while the boundary of the striatal interstitial space is unclear, and many vacuoles can be seen throughout the striatum. The prefrontal cortex in PD + BCCAO 2 weeks group showed that a large number of neurons in the prefrontal cortex were shrunken, the staining of the cells was deepened, the boundary between the nucleus and cytoplasm was unclear, and the cells were loosely arranged. (b) Representative images of Nissl staining in prefrontal cortex and striatum of each group. Dark blue represents Nissl bodies. Nissl bodies are large and numerous, indicating that nerve cells have a strong function of synthesizing proteins; on the contrary, when nerve cells are damaged, the number of Nissl bodies will decrease or even disappear. The numbers of Nissl‐stained neurons in prefrontal cortex and striatum were significantly decreased in the PD + BCCAO 2 weeks group. Scale bars represent 50 μm.

**Figure:**
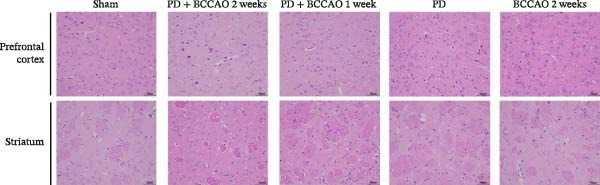
(a)

**Figure:**
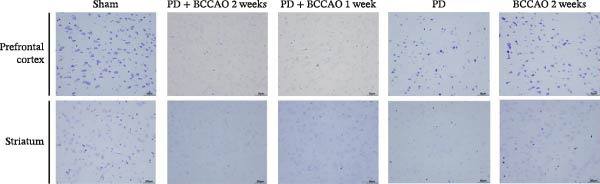
(b)

We apologize for this error.

