# Supplementary figures and images for: Chronic Cerebral Hypoperfusion Aggravates Parkinson's Disease Dementia-Like Symptoms and Pathology in 6-OHDA-Lesioned Rat through Interfering with Sphingolipid Metabolism
Source: Oxid Med Cell Longev. 2022 Aug 8;2022:5392966. doi: 10.1155/2022/5392966 (PMC9377946; doi:10.1155/2022/5392966)

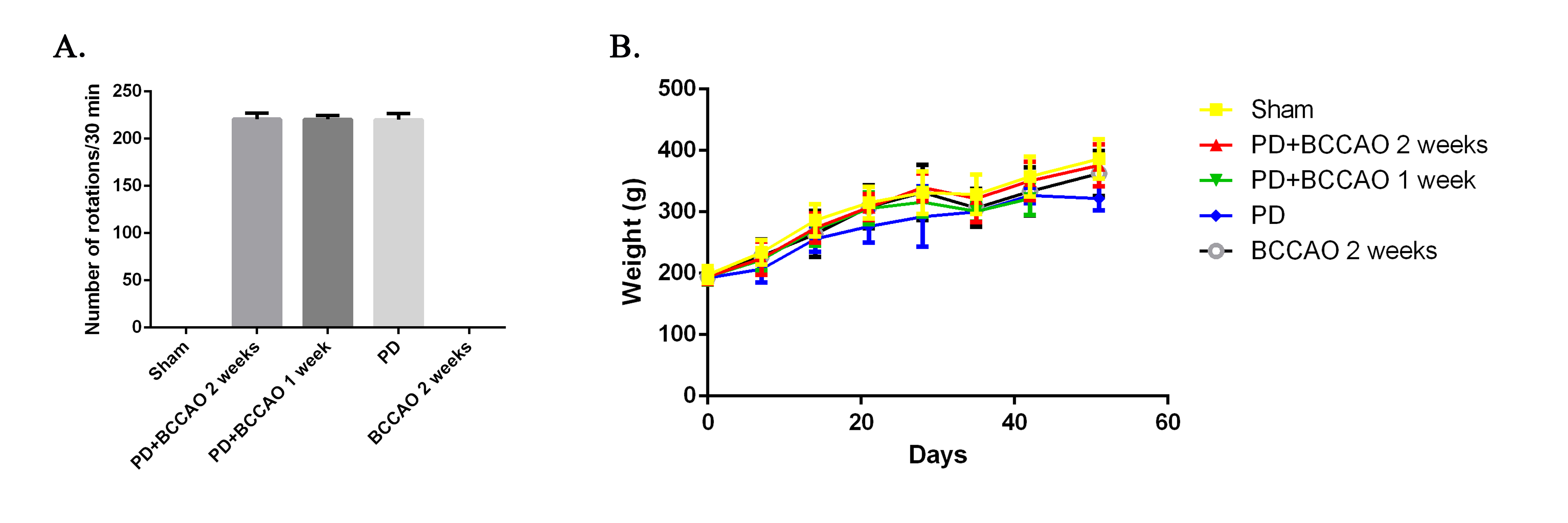

Supplement: Supplementary Materials — Supplemental Figure 1: (A) the number of rotations of each group in apomorphine-induced rotation tests. (B) Weight monitoring during the experiment. Supplemental Figure 2: the QC information of samples. (A) Negative ion mode TIC overlay of QC samples. (B) Metabolic ions distribution in negative ion mode. (C) Positive ion mode TIC overlay of QC samples. (D) Metabolic ions distribution in positive ion mode. (E) PCA analysis of all identified metabolic ions. (F) Correlation analysis of all identified metabolic ions. [file 5392966.f1.zip › S Figure1-0710 (1).jpg]

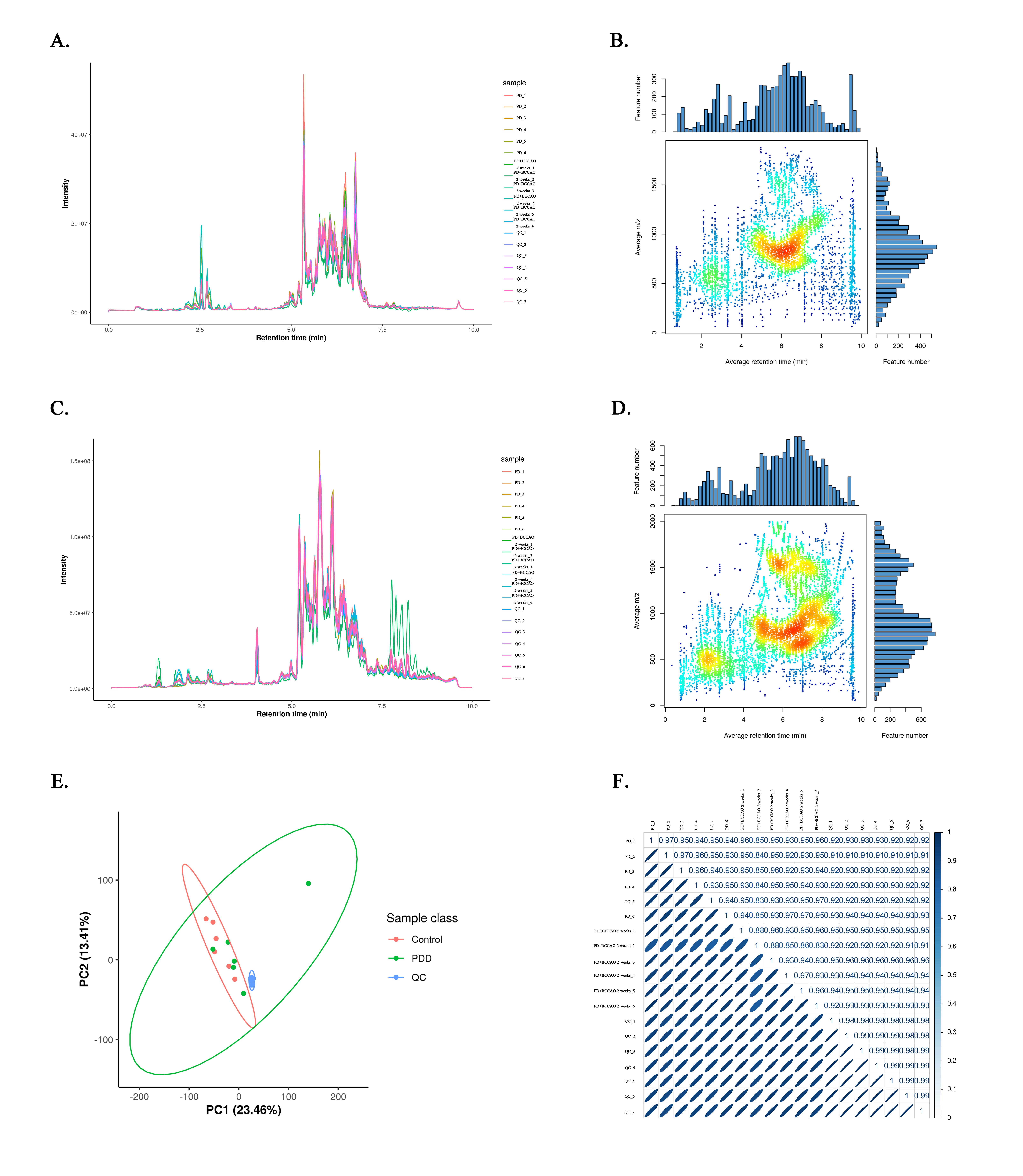

Supplement: Supplementary Materials — Supplemental Figure 1: (A) the number of rotations of each group in apomorphine-induced rotation tests. (B) Weight monitoring during the experiment. Supplemental Figure 2: the QC information of samples. (A) Negative ion mode TIC overlay of QC samples. (B) Metabolic ions distribution in negative ion mode. (C) Positive ion mode TIC overlay of QC samples. (D) Metabolic ions distribution in positive ion mode. (E) PCA analysis of all identified metabolic ions. (F) Correlation analysis of all identified metabolic ions. [file 5392966.f1.zip › S figure2 (1).jpg]
